# Whole Brain Radiotherapy Plus Concurrent Chemotherapy in Non-Small Cell Lung Cancer Patients with Brain Metastases: A Meta-Analysis

**DOI:** 10.1371/journal.pone.0111475

**Published:** 2014-10-27

**Authors:** Hong Qin, Feng Pan, Jianjun Li, Xiaoli Zhang, Houjie Liang, Zhihua Ruan

**Affiliations:** Department of Oncology, Southwest Hospital, the Third Military Medical University, Chongqing, PR China; H. Lee Moffitt Cancer Center, United States of America

## Abstract

**Objective:**

The aim of the present meta-analysis is to evaluate the response rate, median survival time (MST) and toxicity in patients with brain metastases (BM) originating from non-small cell lung cancer (NSCLC) and who were treated using either whole brain radiotherapy (WBRT) plus concurrent chemotherapy or WBRT alone.

**Methods:**

PubMed, EMBASE, Web of Science, The Cochrane Library, clinical trials and current controlled trials were searched to identify any relevant publications. After screening the literature and undertaking quality assessment and data extraction, the meta-analysis was performed using Stata11.0 software.

**Results:**

In total, six randomized controlled trials (RCT) involving 910 participants were included in the meta-analysis. The results of the analysis indicate that WBRT plus concurrent chemotherapy was more effective at improving response rate (RR = 2.06, 95% CI [1.13, 3.77]; P = 0.019) than WBRT alone. However, WBRT plus concurrent chemotherapy did not improve median survival time (MST) (HR = 1.09, 95%CI [0.94, 1.26]; P = 0.233) or time of neurological progression (CNS-TTP) (HR = 0.93, 95%CI [0.75, 1.16]; P = 0.543), and increased adverse events (Grade≥3) (RR = 2.59, 95% CI [1.88, 3.58]; P = 0.000). There were no significant differences in Grade 3–5 neurological or hematological toxicity between two patient groups (RR = 1.08, 95%CI [0.23, 5.1]; P = 0.92).

**Conclusion:**

The combination of chemotherapy plus WBRT in patients with BM originating from NSCLC may increase treatment response rates of brain metastases with limited toxicity. Although the therapy schedule did not prolong MST or CNS-TTP, further assessment is warranted.

## Introduction

Approximately 20% to 40% of patients with cancer develop brain metastases (BM) during their disease course. Patients with solid tumors, such as lung and, breast cancer or melanoma, are at high risk for BM. In particular, it has been estimated that approximately 50% of primary lung cancers develop into BM [1]. Furthermore, non-small cell lung cancer (NSCLC) accounts a large percentage of lung cancer cases. It has also been estimated that 25% to 30% of newly diagnosed NSCLC patients also suffer from brain metastases [2]. NSCLC patients who develop BM often have poor prognoses, severe neurological symptoms, poor quality of life and dismal survival rates. The overall survival time (OS) for NSCLC patients with BM is less than 3–6 months when left untreated [3]; effective treatment options for NSCLC patients with BM are needed urgently.

Whole brain radiotherapy (WBRT) has been the standard therapy for most patients with multiple BM.WBRT can palliate neurological symptoms and control the local disease. However, it has been difficult to eradicate the tumors due to the limitations of radiation therapy. One study reported that one-third of included patients had uncontrollable localized tumors following WBRT treatment and that 50% of patients died of intracranial tumor progression [4]. Systemic chemotherapy has also been used to reduce tumor burden in patients with BM originating from NSCLC. However, the treatment’s effectiveness is limited due to the brain-blood barrier (BBB). Clinical doctors, therefore, faced a dilemma when treating NSLCL patients with BM. Some researchers have suggested that chemical drugs can infiltrate the brain tissue when radiation destroys the BBB, and several clinical trials have indicated that WBRT combined with chemotherapy is not only more effective than WBRT alone, but also improves the response rate and prolongs survival [5]–[7]. Other studies have failed to confirm the efficacy of chemotherapy and suggest that chemotherapy concurrent with WBRT increases the incidence of adverse events and does not benefit NSCLC patients with BM [8]–[10]. The role of chemotherapy concurrent with WBRT for the treatment of patients with BM originating from NSCLC is controversial. We have therefore conducted a meta-analysis assessing the efficacy and safety of chemotherapy combined with WBRT versus treatment with WBRT alone.

## Materials and Methods

### Search strategy

PubMed, EMBASE, the Cochrane Library, Web of Science, clinical trials and current controlled trials were searched to identify relevant studies in the published literature. The search was performed on September 25, 2013, using both Mesh and free text words. The following basic search terms were used: lung neoplasms, lung tumor, lung cancer, brain metastasis, brain neoplasms, radiotherapy and chemotherapy. The search was performed without any language limitations.

### Inclusion criteria

All articles which met the following criteria were eligible: (1) randomized controlled trials (RCT) with voluntarily enrolled patients; (2) patients had histologically or cytologically confirmed NSCLC and had been diagnosed with multiple brain metastases using CT or MRI; (3) the trials compared WBRT plus chemotherapy with WBRT alone; (4) trials did not include patients with chemotherapy contraindications or serious vital organ dysfunction and Karnofsky performance status (KPS) scores ≥70; (5) the analyses included response rate, median survival time (MST), the time to neurological progression (CNS-TTP), adverse events (Grade≥3) or hematological toxicity (Grade≥3); (6) response rate was determined using the Response Evaluation Criteria in Solid Tumors (RECIST) or WHO evaluation criteria on solid tumors. complete remission (CR) was defined as tumor completely disappearing for at least four weeks without any new lesions, partial response (PR) was defined as more than 50% tumor regression for at least for four weeks without new lesions, Progressive disease (PD) was defined as an increase in the sum of the longest diameters (LD) of the target lesions by 25% or higher, using as reference the smallest sum LD recorded since treatment started or the appearance of one or more new lesions. Stabilized disease (SD) was defined as a ≤50% tumor regression or an increase ≤25%. (7) Toxicity was evaluated according to the National Cancer Institute Common Terminology Criteria for Adverse Events.

### Study selection

The eligibility assessment was first performed by screening titles and abstracts and subsequently reviewing the full text of articles. The selection of all studies was performed independently, according to the inclusion criteria, by two reviewers. Disagreement on whether an article should be included was resolved using a third reviewer.

### Data extraction

Two authors independently extracted data from all the eligible studies. When the extracted data were not uniform, consultation was needed to make a final determination. All of the studies included in the analysis contain the following data: first author’s name, published year, type of study, trial phase, country of origin study, percentage of men, performance status, number of patients, average ages, interventions and outcomes.

### Quality assessment

All of the selected studies were evaluated by two reviewers according to the Cochrane Handbook for RCT, based on the following criteria: (1) randomized method; (2) allocation concealment; (3) blinding of participants, personnel and outcome assessment; and (4) intention-to-treat analysis if the trials lost participants to follow-up or if participants quit. Each trial for bias based on the criteria listed above was marked as ‘low risk’, ‘high risk’ or ‘unclear risk’. Trials judged as low risk of bias (i.e. A rating) when all criteria are assessed as low risk; Trials judged as moderate risk of bias (i.e. B rating) when one or more criteria are assessed as unclear risk; Trials judged as high risk of bias (i.e. C rating) when one or more criteria are assessed as high risk.

### Statistical analysis

Statistical analyses were performed using Stata software11.0. Chi-square and I-square tests were used to test the heterogeneity of different studies [11]; no heterogeneity was considered to exist when P>0.1 and I^2^<50%. A fixed-effect model was applied to pool the study results. Significant heterogeneity was found if P<0.1 and I^2^>50%, and a random-effects statistical model was used [12]. Response rate, severe hematological toxicity and advent events were analyzed using dichotomous variables. MST and CNS-TTP were calculated using effect variables.

## Results

### Selection of studies

**Figure 1:**
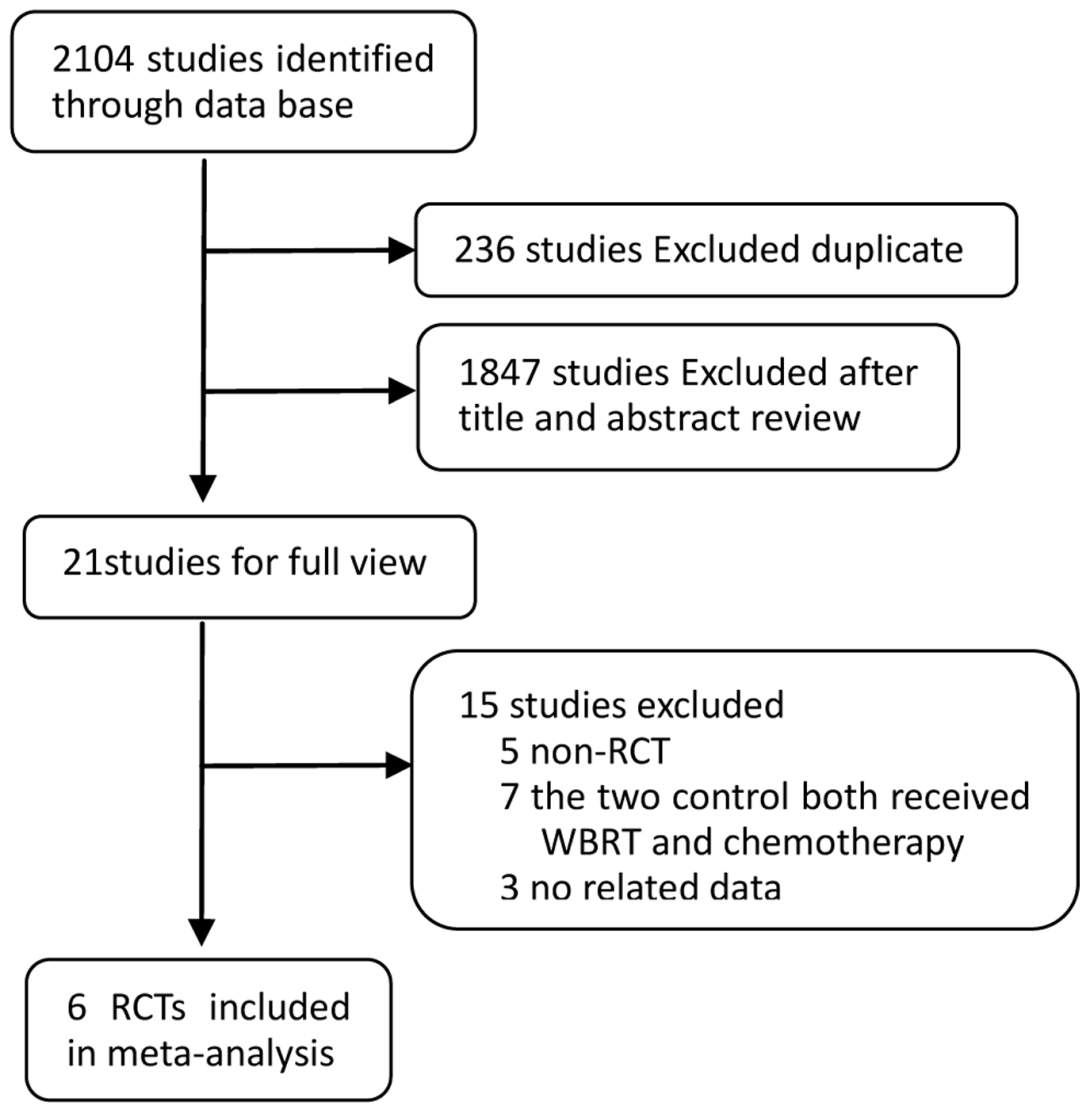
A flow chart on selection included of trials in the Meta-analysis.

In total, we identified 2104 studies that met our selection criteria after searching the relevant databases; 236 of these studies were excluded due to duplication. By verifying related terms in the titles and abstracts, we excluded 1847 irrelevant articles, and another 15 articles were excluded after the full text was read. Finally, six RCTs [10], [13]–[17] were selected for the present meta-analysis. A flowchart depicting the study selection is shown in figure 1.

### General characteristics of included studies

There were 910 patients with BM originating from NSCLC in the six selected RCT trials, with 478 patients having received WBRT concurrent with chemotherapy and 432 patients having received only WBRT; these results are summarized in table 1. Of the six RCTs, three were phase III clinical trials [10], [14], [16], two were phase II studies [15], [17], and one was a study [13] that did not mention a trial phase. The analyzed interventions were WBRT plus chemotherapy and WBRT alone, except in the case of Sperduto, P. W.2013, which compared the combination treatment WBRT, stereotactic radiotherapy (SRS) and chemotherapy with WBRT+SRS treatment. Among all of the included studies, chemotherapy drugs included temozolomide (TMZ), carboplatin, motexafin gadolinium (MGD), chloroethylnitrosoureas and tegafur. TMZ was used in three of the trials. Outcomes included response rate, adverse events, hematological toxicity, median survival time (MST) and time to central nervous system progression (CNS-TTP).

**Table 1:** Characteristics of trials included in the Meta-analysis.

Studies	Country	Trialphase	N(T/C)	Male(T/C,%)	Ages(T/C, years)	ECOG	Interventions		Outcomes	Studyquality
							T	C		
Ushio, Y.1991	Japan	NM	69/31	80/87	57/63	NM	WBRT+methyl-CCNU/ACNU/tegafur	WBRT	Response rate	B
Sperduto, P.W.2013	American	3	40/44	NM	63/64	0–1	WBRT+SRS+TMZ	WBRT+SRS	MST, OS, CNS-TTP, toxicity	B
Mehta, M.P.2009	France	3	279/275	59/55	59/59	0–2	WBRT+MGD	WBRT	MST, CNS-TTP, Adverse event	B
Hassler, M.R.2013	Austria	2	22/13	59/62	69/64	0–2	WBRT+TMZ	WBRT	Response rate, MST, Toxicity, Adverse events	B
Guerrieri, M.2004	Australia	3	21/21	71/71	60/63	0–2	WBRT+ carboplatin	WBRT	MST, Response rate	B
Chua, D.2010	China	2	47/48	64/67	59/62	0–2	WBRT+TMZ	WBRT	MST, CNS-TTP, Toxicity, Adverse event	B

T: treatment group (chemotherapy plus WBRT group); C: control group (WBRT group); NM: not mentioned; ECOG: ECOG/WHO performance status WBRT: Whole brain radiotherapy; TMZ: temozolomide; MST: median survival time; OS: overall survival; CNS: central nervous system; MGD: motexafin gadolinium; TTP: time to progression.

### Methodological quality

In accordance with the recommendations of the Cochrane Handbook for Systematic Reviews, two authors evaluated the eligible studies using the four aspects mentioned above. Four studies [10], [15]–[17] mentioned the use of random allocation, but only two articles discussed the methods [13], [14]. None of the studies performed or reported their allocation concealment and blinding methods. The Hassler, M.R.2013 [15] trial reported follow-up information, but the other studies did not. All of the articles applied the intent-to-treat analysis. The six eligible studies all received B quality scores, as shown in table 1.

### Response rate

**Figure 2:**
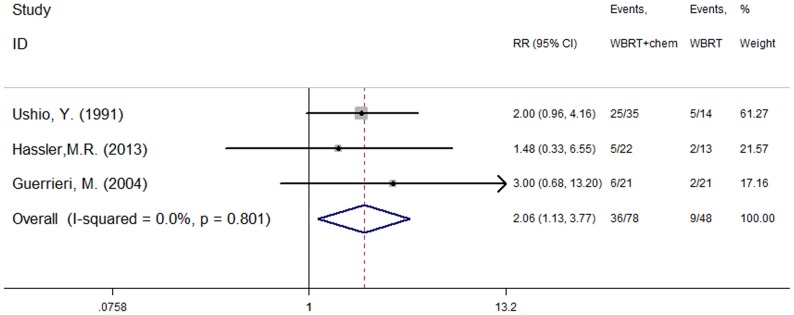
Response rate (P = 0.019).

Three of the included studies [13], [15], [16] reported the efficacy of treatment using WBRT plus concurrent chemotherapy and WBRT alone. Ushio, Y.1991 [13] reported tumor response rates in the WBRT and WBRT plus chemotherapy groups were 36% and 71%, respectively. Hassler, M.R.2013 [15] reported two cases of PR in the WBRT arm, and two CR and three PR cases in the WBRT plus chemotherapy arm. Guerrieri, M.2004 [16] reported response rates were 10% and 29% in the WBRT and WBRT plus carboplatin arms, respectively. There was no heterogeneity (P = 0.801, I^2^ = 0.0%) among the three studies, and as a result, the fixed effect model was used for the meta-analysis. The results indicate that WBRT plus concurrent chemotherapy resulted in superior response rates when compared with WBRT alone (RR = 2.06, 95%CI [1.13, 3.77]; P = 0.019) (figure 2).

### Adverse events

**Figure 3:**
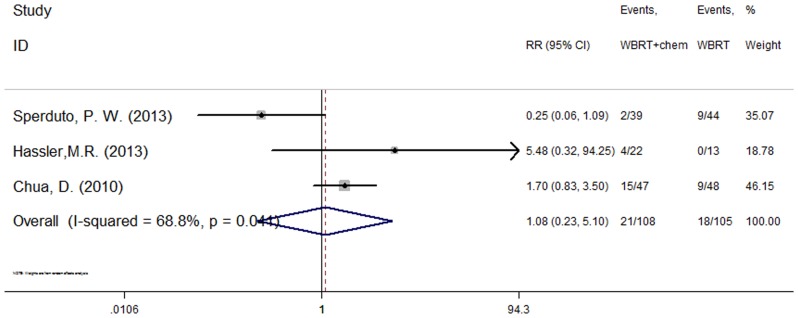
Severe haematological toxicity (P = 0.92).

**Figure 4:**
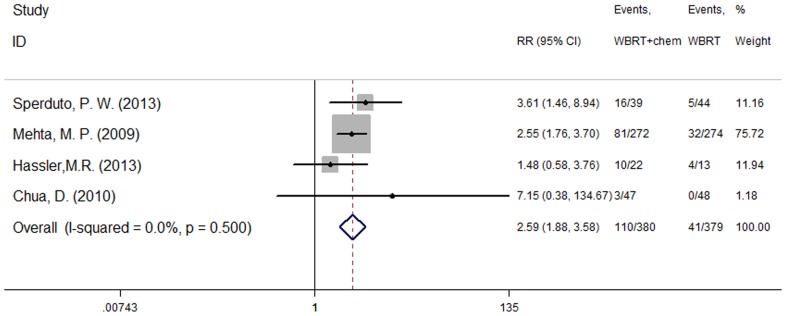
Severe adverse event (P = 0.000).

Three studies [10], [15], [17] reported the occurrence of drug-related hematological toxicity (Grade≥3). A random effects model was used for the meta-analysis of these studies based on the heterogeneity values (P = 0.041, I^2^ = 68.8%). The results indicate no significant difference in hematological toxicity between WBRT plus chemotherapy and WBRT alone (RR = 1.08, 95% CI [0.23, 5.1]; P = 0.92) (figure 3). However, another four studies [10], [14], [15], [17] described adverse events (Grade≥3) and included both hematological and non-hematological toxicity. A fixed effect model was used for the meta-analysis of these studies because heterogeneity did not exist (P = 0.500, I^2^ = 0.0%). The results indicate that the incidence of severe adverse events was higher in the group treated using WBRT concurrent with chemotherapy (RR = 2.59, 95% CI [1.88, 3.58]; P = 0.000) (figure 4).

### Survival

**Figure 5:**
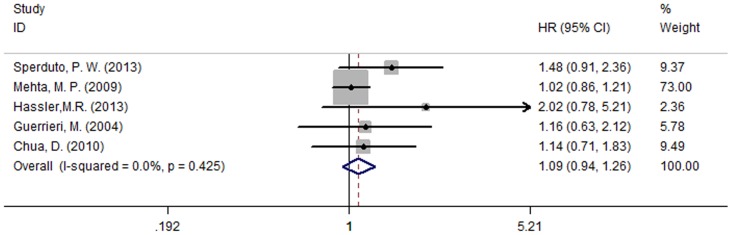
Median survival time (MST) (P = 0.233).

**Figure 6:**
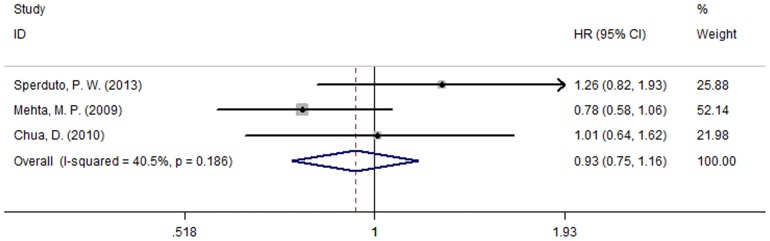
The time to CNS-progression (P = 0.543).

Five of the studies [10], [14]–[17] reported MST for both patient groups; the studies were not heterogeneous (P = 0.425, I^2^ = 0.0%). Analysis using a fixed effect model suggests that in NSCLC patients diagnosed with BM, there was no significant MST difference between those who were treated with chemotherapy and those who were not (HR = 1.09, 95% CI [0.94, 1.26]; P = 0.233) (figure 5). The most meaningful outcome was the time to neurological progression (CNS-TTP). Three studies [10], [14], [17] reported CNS-TTP, and there was no significant heterogeneity between them (P = 0.186, I^2^ = 40.5%); accordingly, a fixed effect model was used for the meta-analysis of CNS-TTP. The results suggest that combining chemotherapy with WBRT could prolong the time of neurological progression (HR = 0.93, 95% CI [0.75, 1.16]; P = 0.543) (figure 6). In conclusion, this meta-analysis suggests that WBRT concurrent with chemoradiotherapy significantly increased response rate and potentially prolonged the time of neurologic progression for patients with BM originating from NSCLC. However, more hypotoxic chemotherapy drugs still need to be explored in future clinical research.

## Discussion

Currently, WBRT is the standard therapy for NSCLC patients whose disease has metastasized to the brain. Several studies have verified that WBRT palliates the neurological symptom associated with BM. However, because radiotherapy doses are limited, the treatment has been unsuccessful at curing malignant lesions. Furthermore, the brain-blood barrier (BBB) prevents the transport of most anticancer agents to the central nervous system and restricts the delivery of drugs to infiltrating BM. These additional barriers restrict the use of chemotherapy for patients with BM. Results of several trails have indicated that chemotherapy combined with WBRT benefits NSCLC patients with BM. Some clinicians found that WBRT could allow chemotherapy drugs to pass through the BBB. Additionally, chemotherapy had the potential to make brain tumor cells more sensitive to radiotherapy. Several studies have indicated that WBRT plus concurrent chemotherapy is playing an increasing role in the treatment of BM. Mehta, M. P. 2009 [14] reported that treatment using motexafin gadolinium (MGd) improved the neurologic progression interval when compared with WBRT alone (15 months vs.10 months). Verger, E. 2005 [18] reported results of patients who received the combination treatment of WBRT with TMZ, noting that they exhibited good tolerance and significantly better progression-free survival of BM at 90 days (54% vs. 72%; P = 0.03). Nonetheless, some studies have suggested that WBRT had a minor effect on promoting chemotherapy drugs across the BBB. Adding chemotherapy to WBRT treatment did not confer any benefits to the patients, but did increase the incidence of adverse events. For instance, Neuhaus, T.2009 [19] reported that concurrent radiochemotherapy (WBRT+topotecan) did not achieve significant curative effects in patients with lung cancer.

A total of six RCTs were included in present meta-analysis. The response rate was significantly improved in patients treated with WBRT plus chemotherapy. However, WBRT plus chemotherapy did not improve MST and CNS-TTP for patients with malignant lesions. WBRT plus chemotherapy increased the incidence of adverse reactions, such as asthenia, fatigue, nausea, vomiting, infection, thrombocytopenia, anemia and neutropenia, but there were no significant differences in the rates of severe hematological toxicity. Each group of patients in the Sperduto, P. W.2013 study received SRS, which might have influenced the final outcomes of the meta-analysis; therefore, we extracted these data and re-analyzed MST and CNS-TTP. Although no significant statistical differences were found, treatment with both WBRT with concurrent chemotherapy tended to prolong MST and CNS-TTP (MST: HR = 1.06, 95% CI [0.91, 1.23], P = 0.462; CNS-TTP: HR = 0.84, 95% CI [0.65, 1.08], P = 0.183).

Researchers hold the opinion that the favorable therapeutic effects of combining chemotherapy with WBRT depend on the drugs crossing the BBB [20]. Temozolomide (TMZ) protocols have been recommended for the favorable distribution of the chemotherapy drug through the BBB and to achieve an effective concentration in the brain tissue [21]. A number of trials have shown that TMZ was well tolerated by patients with BM and achieved high release rates [22], [23]. By contrast, some chemotherapy drugs, such as etoposide and cisplatin, had difficulty reaching the intracranial environment because of the BBB. Those agents did not improve response rates and only increased the incidence of neurological toxicity [8]. Due to a lack of response rates and CNS-TTP data, we did not analyze the differences between TMZ and non-TMZ treatments.

Our present systematic review suggests that the combination of chemotherapy and WBRT does not obviously improve MST and CNS-TTP. Moreover, we found a tendency toward prolonged CNS-TTP intervals in the concurrent chemoradiotherapy group.

Our results suggest that the combination of chemotherapy and WBRT significantly increased adverse events of Grade 3 or higher, although there was insufficient evidence to indicate that the treatment resulted in severe nervous-system toxicity. Sperduto, P. W.2013 [10] reported that rates of Grade 3–5 toxicity for WBRT/SRS and WBRT/SRS/TMZ were 11% and 41%, respectively. However, most of the adverse events were fatigue, dehydration and other aspecific symptoms. Chua, D.2010 [17] reported that three patients suffered adverse events (≥Grade 3), and none of these events were related to neurologic toxicity.

Meta-analysis is based on the results of published articles and several steps of integration; thus, certain biases are inevitable. Also, 6 studies included in the meta-analysis were published in the period 1991–2013. Although the dose of WBRT for the treatment of multiple brain metastases did not change during this 22-year period, more precisely delineated target regional and advanced equipment would affect the effectiveness of treatment as technology advances. In addition, as more and more researchers focused their attention on the investigation of targeted drug combine with WBRT in the treatment of multiple brain metastases, and many chemoradiotherapy-related studies were retrospective study and single-arm study, limited RCT were included in this study. Furthermore, the methods of randomization, allocation concealment, and blinding in most of the included studies are not clear. As a result, the quality of the 6 RCTs was not high. The different strategies used to divide groups and the dose of WBRT and chemotherapy drugs were not clear; these procedures may have influenced the final outcomes. More high quality and large scale trials are necessary to confirm the efficacy and safety of WBRT plus concurrent chemotherapy for the treatment of patients with BM originating from NSCLC.

In conclusion, this meta-analysis reports that concurrent chemoradiotherapy significantly increased response rates and had the potential to prolong neurologic progression time NSCLC patients with BM. However, additional future clinical research is needed to explore the use of more hypotoxic chemotherapy drugs.

## Supporting Information

Checklist S1 PRISMA Checklist.
(DOC)

Figure S1 PRISMA Flow Diagram.
(DOC)
