# Supplementary material for: Whole Brain Radiotherapy Plus Concurrent Chemotherapy in Non-Small Cell Lung Cancer Patients with Brain Metastases: A Meta-Analysis
Source: PLoS One. 2014 Oct 27;9(10):e111475. doi: 10.1371/journal.pone.0111475 (PMC4210217; doi:10.1371/journal.pone.0111475)
Supplement: Figure S1 — PRISMA Flow Diagram. (DOC) [file pone.0111475.s002.doc]

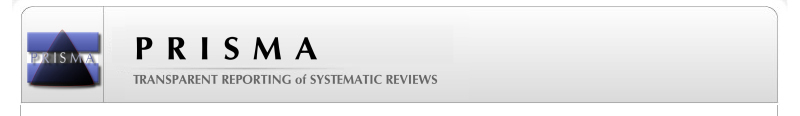
**PRISMA 2009 Flow Diagram**

**Screening**

**Included**

**Eligibility**

**Identification**

Records identified through database searching
(n =2104 )

Additional records identified through other sources
(n =0 )

Records after duplicates removed
(n = 236 )

Records screened
(n = 1868 )

Records excluded
(n = 1847 )

Full-text articles assessed for eligibility
(n = 21 )

Full-text articles excluded, with non-RCT, no related data and both received WBRT and chemotherapy (n =15 )

Studies included in qualitative synthesis
(n = 6 )

Studies included in quantitative synthesis (meta-analysis)
(n = 6 )
